# Initial management strategies for right-sided malignant colonic obstruction: a systematic review and bayesian network meta-analysis

**DOI:** 10.1186/s12957-026-04303-9

**Published:** 2026-04-02

**Authors:** Daichi Kitaguchi, Antonello Forgione, Chiara Innocenzi, Yuchuan Yang, Federico Espínola Schulze, Ian Dox, Mariano Giménez, Tatsuya Oda, Jacques Marescaux

**Affiliations:** 1 Research Institute against Digestive Cancer (IRCAD), 1 place de l’Hôpital, Strasbourg Cedex, 67091 France; 2 Department of Gastrointestinal and Hepatobiliary and Pancreatic Surgery, Institute of Medicine, University of Tsukuba, Tsukuba, Ibaraki Japan; 3 Department of Women, Children, and Public Health Sciences, Fondazione Policlinico Universitario Agostino Gemelli IRCCS, Rome, Italy

**Keywords:** Colorectal neoplasms, Colonic neoplasms, Intestinal obstruction, Stents, Surgical stomas

## Abstract

**Background:**

Right-sided malignant colonic obstruction (RMCO) remains challenging, as the evidence is more limited than for left-sided disease. Although primary resection is the standard approach, emergency surgery carries higher perioperative risks, increasing interest in bridge-to-surgery (BTS) strategies. However, their optimal role remains uncertain. We conducted a systematic review and Bayesian network meta-analysis to compare initial management strategies for RMCO and evaluate short- and long-term outcomes.

**Methods:**

A comprehensive search of PubMed, Embase, and the Cochrane Library was performed, with a last update in October 2025. Comparative studies including patients with potentially curable RMCO who underwent either immediate surgery or a BTS approach (stent, decompression stoma, or decompression tube) were eligible. A Bayesian random-effects network meta-analysis was conducted. The primary outcome was overall postoperative morbidity; secondary outcomes included postoperative mortality, stoma formation after resection, and overall survival (OS) rate.

**Results:**

A total of 18 retrospective non-randomized cohort studies including 7,205 patients met the inclusion criteria. Stenting was associated with a significantly lower postoperative mortality as compared to immediate surgery (6.6 vs. 0.9%; RR: 0.33; 95% CrI: 0.10–0.75) and with a significantly lower rate of stoma formation after resection (11.8 vs. 2.3%; RR: 0.41; 95% CrI: 0.21–0.78). No significant differences were observed among treatment strategies in overall postoperative morbidity or OS rate.

**Conclusions:**

Stenting as a bridge to surgery (BTS) was associated with a lower postoperative mortality and a lower rate of stoma formation as compared to immediate surgery. These findings suggest that stenting may serve as a viable alternative for patients at high risk of postoperative mortality.

**Supplementary Information:**

The online version contains supplementary material available at 10.1186/s12957-026-04303-9.

## Introduction

Acute malignant colonic obstruction is one of the most common emergency presentations of colorectal cancer, and its initial management remains complex [1]. Right-sided malignant colonic obstruction (RMCO) accounts for approximately 20 to 30%of patients with colorectal cancer who present with obstruction [2], is particularly challenging as the available evidence is more limited than for left-sided obstruction. RMCO also differs anatomically, with a more distensible cecum and a thinner colonic wall, both increasing the risk of perforation, and with reduced stent accessibility due to the longer distance and tortuous anatomy [3]. Traditionally, primary resection with anastomosis, with or without a defunctioning ileostomy has been the preferred treatment for RMCO [4]. However, emergency resection has been suggested to be associated with significantly higher rates of mortality and morbidity, as well as a poorer long-term survival, as compared to elective surgery [5, 6]. Such concerns have generated a growing interest in a bridge-to-surgery (BTS) approach as a potentially more favorable alternative.

According to the 2020 update of the European Society of Gastrointestinal Endoscopy (ESGE) guidelines, colonic stenting as a BTS approach to left-sided malignant obstruction is strongly recommended as an alternative to emergency resection, provided that it is discussed within a shared decision-making process. This recommendation is supported by high-quality evidence [7]. In contrast, for RMCO, ESGE only suggests to consider stenting as a BTS approach, reflecting a weak recommendation based on low-quality evidence [7]. In clinical practice, not only does BTS for RMCO include colonic stenting but it also comprises other decompression strategies, such as decompression stoma formation and decompression tube placement, although the evidence supporting such approaches is similarly limited. Since direct head-to-head comparisons among the different BTS options are limited, a network meta-analysis is required to compare their relative effectiveness.

Consequently, we aimed to conduct a systematic review and network meta-analysis to compare the available initial management strategies for RMCO, including immediate surgery and various BTS approaches, and to clarify their relative safety, short-term surgical outcomes, and long-term survival.

## Materials and methods

### Literature search

This systematic review was conducted according to the Preferred Reporting Items for Systematic Reviews and Meta-Analyses for Network Meta-Analyses (PRISMA-NMA) guidelines [8]. The protocol was prospectively registered in the International Prospective Register of Systematic Reviews (PROSPERO; CRD420251234425) prior to data extraction and is available at: https://www.crd.york.ac.uk/PROSPERO/view/CRD420251234425. A comprehensive literature search was conducted in PubMed, Embase, and the Cochrane Library.

### Search strategy

A core search strategy was developed using a combination of free-text terms and controlled vocabulary related to right-sided malignant colonic obstruction (RMCO), as well as terms describing the initial management strategies of interest, including surgery, stent, stoma, and decompression tube. The base search string was the following: ((“Colorectal Neoplasms” OR “colon cancer” OR “colorectal cancer”) AND (obstruction* OR obstructive OR ileus) AND (“right-sided” OR “right sided” OR “right and” OR proximal OR cecum OR “ascending colon”) AND ((stent* OR “self-expandable metallic stent” OR SEMS OR “bridge to surgery”) OR (stoma OR ileostom* OR colostom* OR diversion) OR decompression OR (surgery OR colectomy OR resection OR “right hemicolectomy”)) AND (randomized OR random* OR trial OR comparative OR compare* OR cohort OR retrospective OR groups OR propensity OR “case-control” OR observational)). This core strategy was subsequently tailored to each database by incorporating controlled vocabulary (e.g., MeSH in PubMed and Emtree in Embase) and applying database-specific syntax. Complete search strategies for all databases were presented in Supplementary Table 1. The search was restricted to human studies published in English. The final search update was conducted in October 2025.

#### Eligibility criteria

Studies were included if they met all the following criteria:

- Comparative design, including randomized controlled trials (RCTs) and non-randomized studies,
- Adult patients with RMCO treated with curative intent (palliative cases were excluded),
- Reporting at least one surgical or oncologic outcome following initial management with either immediate surgery or BTS approaches, including stent, stoma, or decompression tube,
- Sufficient data available to calculate effect estimates.

Studies were excluded if they were study protocols, unpublished studies, non-original reports (e.g., letters, editorials, comments, conference abstracts, corrections, or replies), studies without extractable data, or review articles.

### Data extraction

Two authors independently performed data extraction. Any discrepancies were resolved through discussion and consensus. Missing data were reported as presented in the original studies and were not imputed or estimated. When overlapping cohorts were identified, the most comprehensive or recent dataset was included to prevent duplication. For observational studies, event counts from propensity score-matched (PSM) cohorts were preferentially extracted when available. Otherwise, unadjusted event counts were used to ensure consistency and comparability across studies within the network meta-analysis framework.

### Study selection

All articles identified during the initial search were independently screened based on their titles and abstracts according to the predefined eligibility criteria. The authors proceeded with a full-text review of studies, which met the eligibility criteria in the initial screening to determine final inclusion.

### Quality assessment

All RCTs were assessed for risk of bias using the revised Cochrane tool for randomized trials (RoB 2) [9]. In contrast, non-randomized studies were evaluated using the Risk of Bias in Non-randomized Studies of Interventions tool (ROBINS-I) [10]. Two authors assessed the quality of all included studies, resolving disagreements through discussion.

### Outcomes

The primary outcome was overall postoperative morbidity. Secondary outcomes included postoperative mortality, stoma formation after resection, and overall survival (OS) rate. When multiple time points were reported for OS, the longest available follow-up was extracted.

### Statistical analysis

Network meta-analyses were conducted using a Bayesian random-effects consistency model to compare all initial management strategies for RMCO. Treatment effects were synthesized using raw event count data to ensure consistency across studies within the network meta-analysis framework. Non-informative normal priors were assigned to treatment effects, and a vague prior was applied to the between-study heterogeneity parameter. Between-study heterogeneity (τ²) was estimated within the Bayesian framework. Effect estimates were reported as risk ratios (RRs) with 95% credible intervals (CrIs). Treatment ranking was evaluated using surface under the cumulative ranking curve (SUCRA) values and visualized with Litmus Rank-O-Gram plots [11]. Inconsistency between direct and indirect evidence was assessed using node-splitting models, with P values < 0.05 considered indicative of inconsistency. To assess the transitivity assumption, potential effect modifiers, including age, sex, American Society of Anesthesiologists physical status (ASA-PS), and stage IV disease, were examined across treatment comparisons based on clinical relevance and data availability.

For continuous outcomes reported only as medians with ranges or interquartile ranges, the corresponding means and standard deviations (SDs) were approximated using the methods proposed by Wan et al. and Luo et al. [12, 13]. Sensitivity analyses were performed to examine the robustness of the findings by excluding studies which could contribute to heterogeneity. All network meta-analyses were performed using Meta Insight v6.4.0 [14]. Bayesian statistical computations were conducted using R (packages *gemtc*, *BUGSnet*, and *bnma*), which implemented Markov chain Monte Carlo methods via the JAGS engine. Convergence was assessed using Gelman convergence assessment plots, trace plots, and posterior density plots. Detailed model settings and convergence diagnostics are provided in Supplementary Table 2.

## Results

### Study selection

**Fig. 1:**
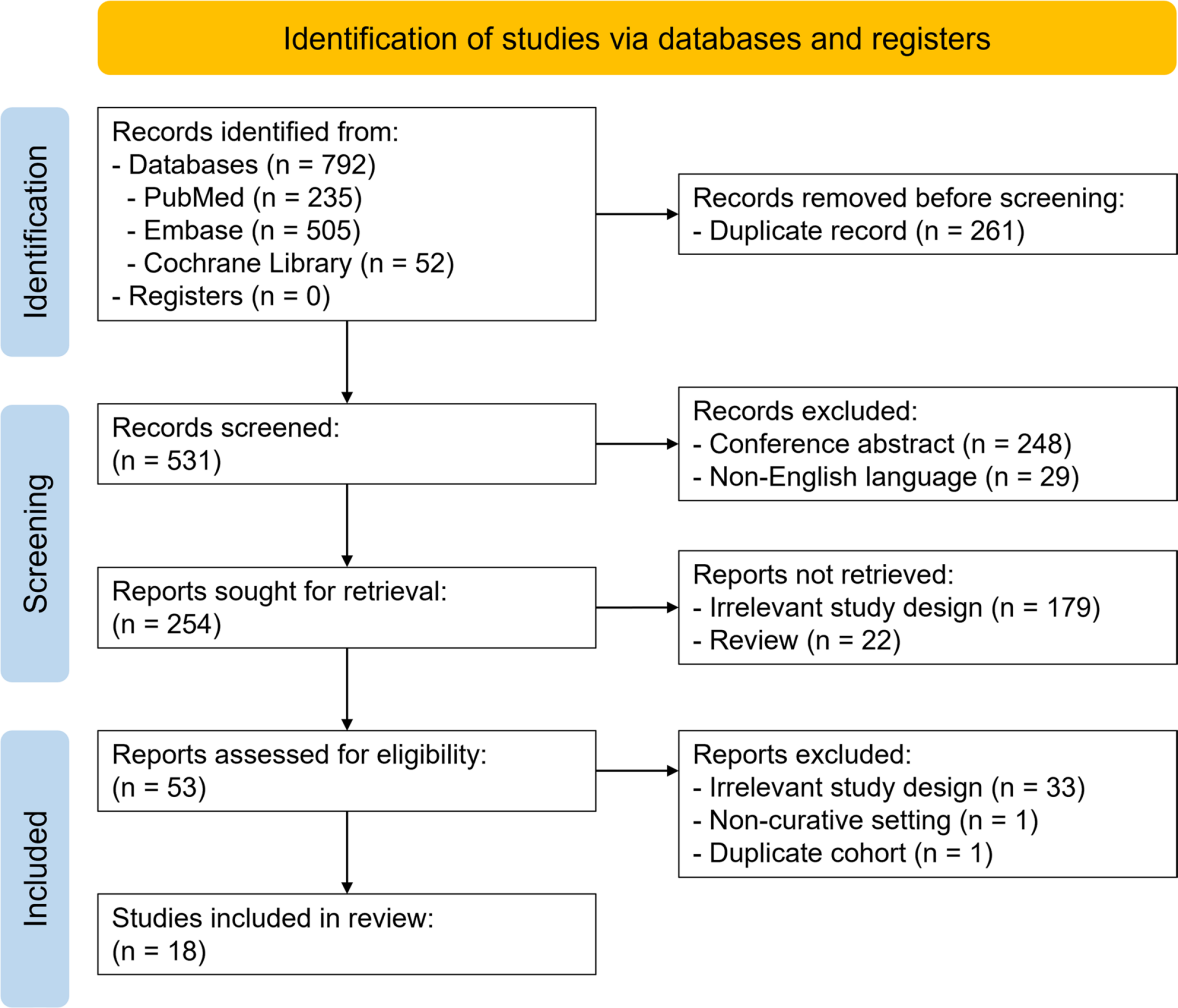
PRISMA flow diagram of study selection

Figure 1 aimed to illustrate the PRISMA flow of study selection. A total of 792 records were identified from the databases (235 from PubMed, 505 from Embase, and 52 from the Cochrane Library). After removing 261 duplicates, 531 records remained. Of these, 248 conference abstracts and 29 non-English articles were excluded, leaving 254 records for title and abstract screening. During this phase, 179 articles were excluded for being irrelevant study design and 22 for reviews. A total of 53 full-text articles were assessed for eligibility. Of these, 33 were excluded due to irrelevant study design, one due to a non-curative setting, and one due to a duplicate cohort. Ultimately, 18 non-randomized studies were included in this systematic review [15–32], and no RCTs were identified.

### Study characteristics

Table 1 summarizes the characteristics of the 18 included studies, comprising 6 single-center and 12 multicenter retrospective cohort studies. Six studies used either propensity score matching (PSM) or multivariable analysis (MVA) to reduce confounding.

**Table 1:** Summary of study characteristics of included studies

Author/Year	Country	Study design	Centers	Comparison	Sample size	Reported outcomes	Clinical success	BTS-related complications	Follow-up*
Amelung 2016	Netherlands	Retrospective	NDB	Stent vs. Stoma vs. IS	44 vs.42 vs. 1,774	Short-term outcomes	NR	NR	NA
Amelung 2017	Netherlands	PSM	NDB	Stent vs. IS	22 vs. 88	Short-term outcomes	19/22 (86%)	0/22 (0%)	NA
Boeding 2023	Netherlands	Retrospective	NDB	Any BTS vs. IS	26 vs. 463	Mortality and OS	NR	NR	67.2
Hotta 2012	Japan	MVA	Single	Stoma vs. Tube vs. IS	2 vs. 8 vs. 28	Short- and long-term outcomes	NR	NR	60
Huang 2024	China	Retrospective	Single	Stent vs. IS	35 vs. 60	Short- and long-term outcomes	31/35 (89%)	2/35 (6%)	44.5
Ji 2017	Korea	Retrospective	3	Stent vs. IS	14 vs. 25	Short- and long-term outcomes	14/16 (88%)	0/16 (0%)	17
Kim 2023	Korea	Retrospective	4	Stent vs. IS	17 vs. 28	Short- and long-term outcomes	NR	NR	53.4
Kye 2016	Korea	Retrospective	7	Stent vs. IS	25 vs. 49	Short- and long-term outcomes	NR	NR	40.8
Li 2020	China	Retrospective	Single	Stent vs. IS	35 vs. 72	Short- and long-term outcomes	34/35 (97%)	0/35 (0%)	35
Lockhorst 2025	Netherlands	Retrospective	Single	Any BTS vs. IS	30 vs. 32	Short- and long-term outcomes	NR	NR	19
Morita 2019	Japan	Retrospective	19	Stent vs. IS	28 vs. 40	Short-term outcomes	27/28 (96%)	0/28 (0%)	NA
Park 2023	Korea	Retrospective	6	Stent vs. Stoma	42 vs. 30	Short- and long-term outcomes	40/42 (95%)	2/42 (5%)	32
Rosander 2021	Sweden	MVA	NDB	Stoma vs. IS	70 vs. 681	Short- and long-term outcomes	NA	NR	18.6
Sakamoto 2020	Japan	PSM	NDB	Stent vs. IS	1,500 vs. 1,500	Short-term outcomes	NR	NR	NA
Suzuki 2019	Japan	MVA	Single	Stent vs. Tube	19 vs. 21	Short- and long-term outcomes	17/19 (90%) 18/21 (86%)	2/19 (11%) 3/21 (14%)	36.2
Takahashi 2024	Japan	Retrospective	Single	Stent vs. Tube	8 vs. 20	Short- and long-term outcomes	NR	0/8 (0%) 0/20 (0%)	31.9
van den Berg 2014	Netherlands	Retrospective	2	Stent vs. IS	16 vs. 17	Short-term outcomes	13/16 (81%)	0/16 (0%)	NA
Zeng 2021	China	PSM	3	Stent vs. IS	98 vs. 196	Short- and long-term outcomes	93/98 (95%)	1/98 (1%)	49

*Median months

China People’s Republic of China, Korea Republic of Korea, *PSM *Propensity score matching, *MVA *Multivariable analysis, *NDB *National database, *BTS *Bridge to surgery, *Stent *Stent as BTS, *Stoma *Decompression stoma as BTS, *Tube *Nasal/transanal decompression tube as BTS, *IS *Immediate surgery without BTS, *OS *Overall survival, *NR *Not reported, *NA *Not applicable

A total of 7,205 patients were included: 5,053 patients underwent immediate surgery without BTS (IS group), 1,903 patients underwent stenting as a BTS approach (stent group), 144 patients underwent decompression stoma formation as a BTS approach (stoma group), 49 patients underwent nasal or transanal decompression tube placement as a BTS approach (decompression tube group), and 56 patients underwent either stent, stoma, or tube as a BTS approach (any-BTS group). Regarding geographic distribution, 12 studies were conducted in Asia (five in Japan, four in South Korea, and three in China), and 6 studies were conducted in Europe (five in the Netherlands and one in Sweden). Notably, studies involving a decompression tube were exclusively conducted in Japan, reflecting its limited adoption outside this region.

The rate of clinical success, defined as the relief of obstructive symptoms within 24 to 48 h after stent or decompression tube placement, was reported in nine studies. Clinical success was achieved in 93% of patients in the stent group (288/311) and 86% in the decompression tube group (18/21). BTS-related complications, including perforation and migration, were reported in 10 studies. The complication rate was 2% in the stent group (7/319) and 7% in the decompression tube group (3/41). Thirteen studies reported both short- and long-term outcomes, and the mean of the reported median follow-up durations was 38.8 months.

Additional baseline patient characteristics of the included studies are summarized in Supplementary Table 3. The distribution of potential effect modifiers, including age, sex, ASA-PS, and stage IV disease, across treatment nodes is presented in Supplementary Table 4. These characteristics were generally comparable across treatment groups, supporting the transitivity assumption.

### Bias and quality analysis

**Fig. 2:**
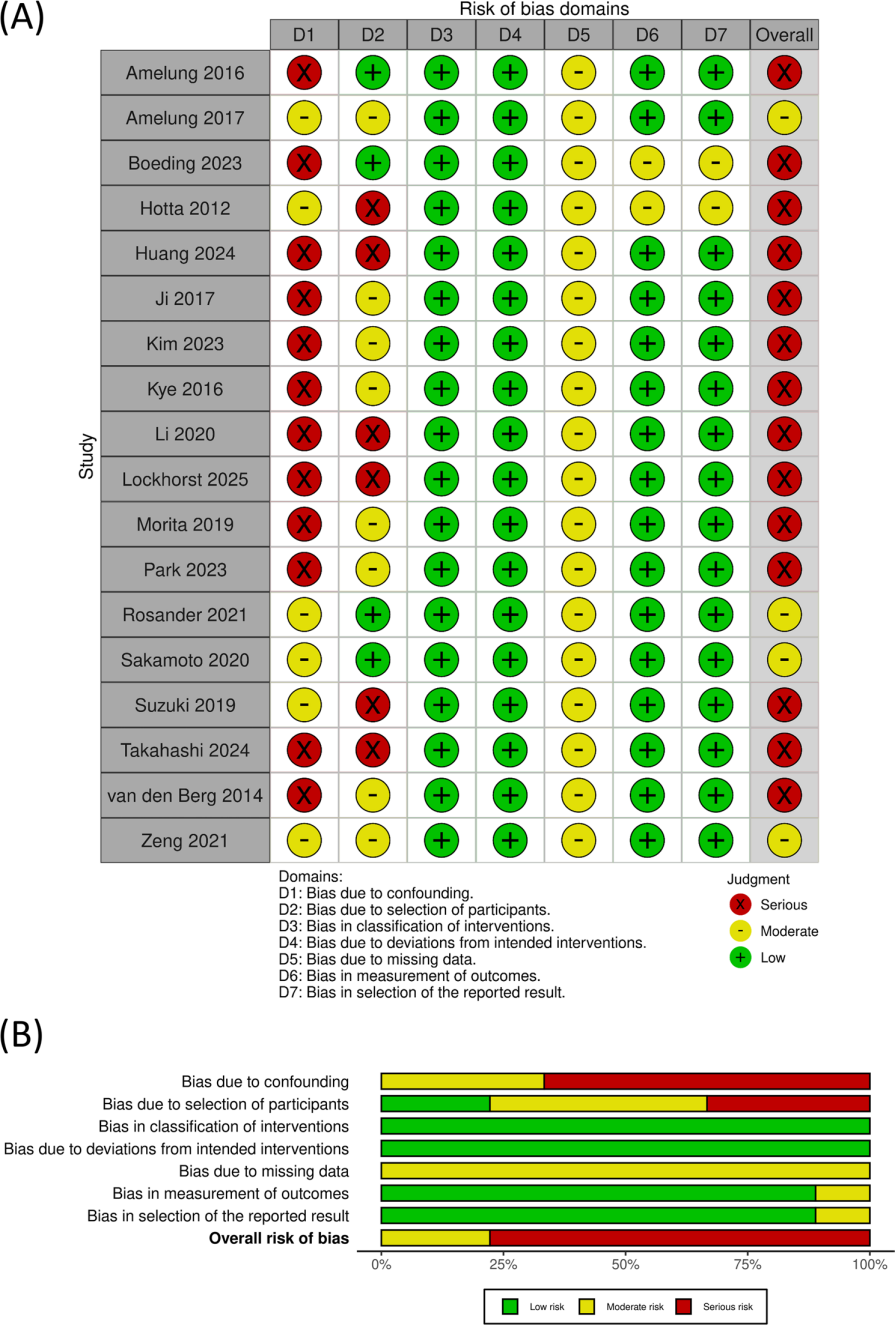
Risk of bias assessment of the included studies using ROBINS-I. **A** Traffic-light plot illustrating the risk of bias for each individual study. **B** Summary plot showing the proportion of risk-of-bias judgments across all studies

The results of the risk-of-bias assessments conducted using ROBINS-I are summarized in Fig. 2. All included studies used a non-randomized retrospective design. Among them, 12 studies which did not use either PSM or MVA were judged to have a serious risk of bias due to confounding. In addition, the 6 studies conducted in a single center were considered to have a serious risk of bias due to the selection of participants. Consequently, 14 studies were rated as having a serious overall risk of bias, whereas the remaining 4 studies were rated as having a moderate overall risk of bias.

### Network geometry

**Fig. 3:**
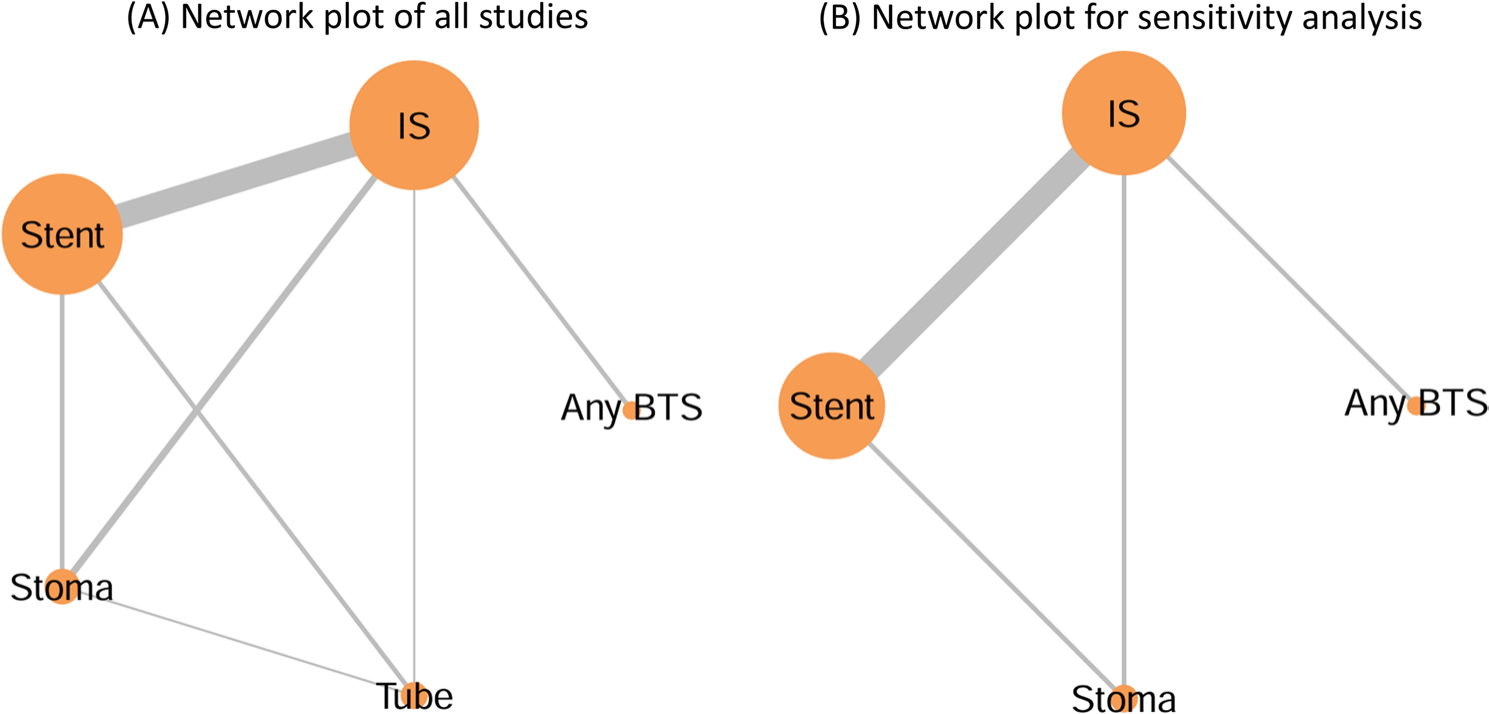
Network plots of treatment comparisons. The size of each node is proportional to the number of patients in that treatment group, and the thickness of each connecting line represents the number of studies reporting that direct comparison. **A** Network plot of all included studies. ts the results of the Bayesian network meta-analysis across all outcomes. For overall postope (**B**) Network plot for the sensitivity analysis excluding the study containing the decompression tube group. (BTS, bridge to surgery; IS, immediate surgery without BTS; Stent, stent as BTS; Stoma, decompression stoma as BTS; Tube, nasal/transanal decompression tube as BTS)

Figure 3 presents the network plots for each outcome. In these plots, the size of each node is proportional to the number of patients in the corresponding treatment group, and the thickness of each connecting line represents the number of studies reporting that direct comparison. Figure 3A shows the network plot of all included studies, whereas Fig. 3B shows the network plot for the sensitivity analysis excluding the study containing the decompression tube group. All treatment nodes were connected within both networks, and both network plots formed closed loops, allowing inconsistency assessment using the node-splitting model. The results of the inconsistency assessment are shown in Supplementary Fig. 1.

### Network meta-analysis results

**Fig. 4:**
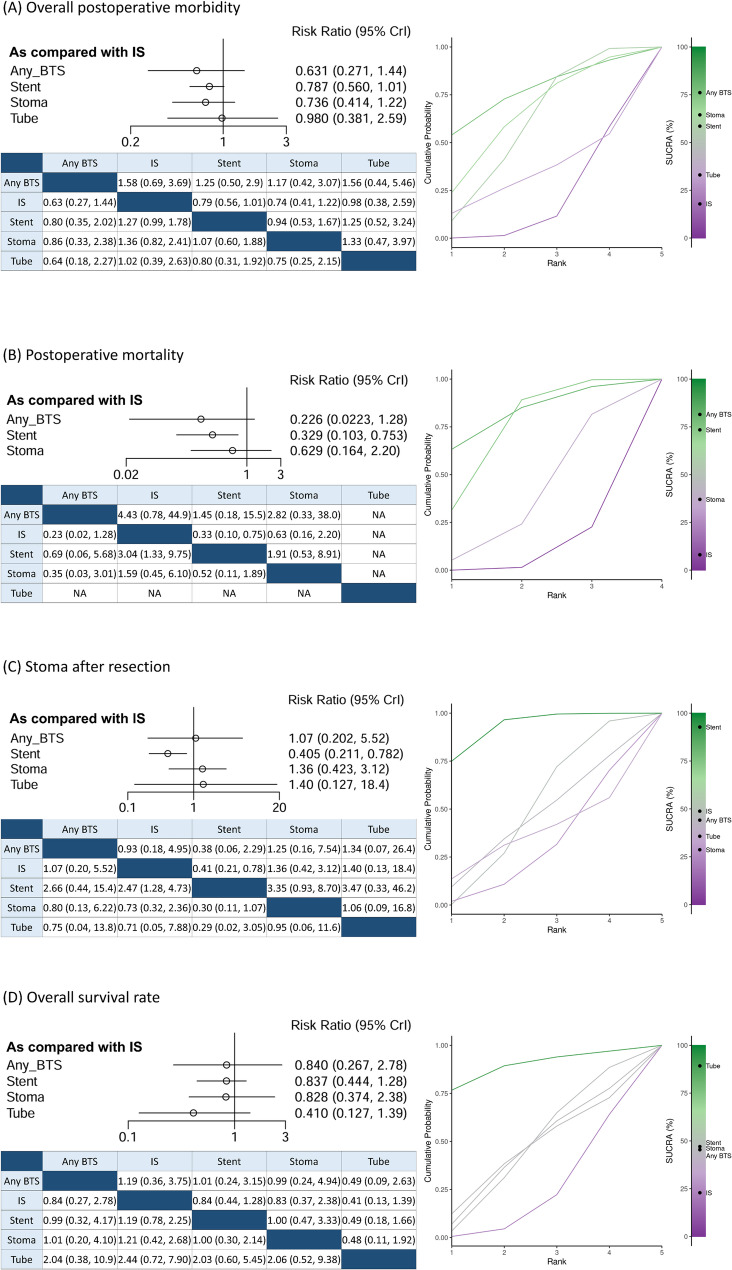
Forest plots, pairwise comparisons, and the SUCRA-based treatment hierarchy derived from the Bayesian network meta-analysis of initial management strategies for potentially curable right-sided malignant colonic obstruction. **A** Overall postoperative morbidity. **B** Postoperative mortality. **C** Stoma formation after resection. **D** Overall survival rate (BTS, bridge to surgery; IS, immediate surgery without BTS; Stent, stent as BTS; Stoma, decompression stoma as BTS; Tube, nasal/transanal decompression tube as BTS; CrI, credible interval; SUCRA, surface under the cumulative ranking curve, where higher SUCRA values indicate better treatment ranking)

Figure 4 presents the results of the Bayesian network meta-analysis across all outcomes.

For overall postoperative morbidity (Fig. 4A), the pooled morbidity rate was 28.6% (1.911/6.678), with no statistically significant differences were observed in any pairwise comparisons. Similarly, no significant differences were observed among the groups in the Bayesian network meta-analysis of severe postoperative morbidity (Clavien–Dindo ≥ grade III), as shown in Supplementary Fig. 2.

For postoperative mortality (Fig. 4B), the overall pooled postoperative mortality rate was 5.1% (368/7,205). The definition of postoperative mortality varied across studies: 8 studies reported 30-day mortality, 3 reported 90-day mortality, 3 reported in-hospital mortality, and 4 did not specify the timeframe. As no postoperative deaths were reported in the decompression tube group, creating a structural zero event which cannot contribute estimable relative effects and may lead to unstable Bayesian model convergence, the decompression tube group was excluded from the mortality network (Fig. 3B). The stent group was associated with a significantly lower postoperative mortality as compared to the IS group (RR: 0.33, 95% CrI: 0.10–0.75), corresponding to absolute mortality rates of 0.9% in the stent group versus 6.6% in the immediate surgery group.

For the rate of stoma formation after resection (Fig. 4C), the stent group was associated with a significantly lower rate of stoma formation as compared to the IS group (RR: 0.41, 95% CrI: 0.21–0.78), with absolute stoma rates of 2.3% versus 11.8%, respectively. However, as shown in Supplementary Fig. 1C1, significant inconsistency was identified in this network. As a result, a sensitivity analysis excluding the decompression tube group (Fig. 3B) was conducted, which resolved the inconsistency (Supplementary Fig. 1C2). Even after this adjustment, the stent group remained associated with a significantly lower rate of stoma formation as compared to the IS group (RR: 0.42, 95% CrI: 0.24–0.83).

For the OS rate (Fig. 4D), no statistically significant differences were observed in any pairwise comparisons.

Treatment rankings based on SUCRA values for all interventions are summarized in Supplementary Table 5. Overall, BTS interventions showed higher SUCRA values than immediate surgery for overall postoperative morbidity, postoperative mortality, and OS rate. For stoma formation after resection, the stent group had the highest SUCRA value among all treatments.

## Discussion

This systematic review and Bayesian network meta-analysis compared immediate surgery without BTS with several BTS approaches, including stenting, decompression stoma, and decompression tube placement, for potentially curable RMCO. Stenting as BTS was associated with a significantly lower postoperative mortality and a significantly lower rate of stoma formation after resection as compared to immediate surgery without BTS. In contrast, overall postoperative morbidity and OS rate were comparable among treatment groups. As compared to left-sided colonic obstruction, immediate surgery tends to be selected more often as the initial management strategy for RMCO. However, such findings suggest that stenting as a BTS approach may represent a reasonable alternative to immediate surgery, especially in patients at a high risk for postoperative mortality, such as the elderly or patients with severe comorbidities.

Among the available BTS options, stenting requires specific clinical consideration. Although the results of this meta-analysis support the use of stenting as BTS for RMCO, a major concern remains the risk of stent-related complications. For left-sided malignant colonic obstruction, two previous RCTs comparing stenting as BTS with immediate surgery were terminated early since the stent arms showed higher complication rates, poor technical success, and increased 30-day morbidity after stent insertion [33, 34]. In the present pooled analysis, the overall rate of stent-related complications, including perforation and migration, was only 2% (7/319), which is substantially lower than the approximately 10% complication rates commonly reported for stenting in left-sided obstruction [35–38]. However, this finding should be interpreted with caution as all included studies were retrospective in design, representing low-quality evidence with a high risk of selection bias; consequently, the observed complication rate may have been underestimated. As a result, multicenter prospective registries or RCTs are necessary to more accurately determine the true incidence of stent-related adverse events in RMCO.

In contrast, decompression stoma represents another BTS option. However, its benefit differs substantially between left- and right-sided obstruction. In left-sided malignant colonic obstruction, Veld et al. reported in a nationwide PSM study that decompression stoma as a BTS approach was associated with a significantly lower postoperative mortality and improved OS as compared to immediate surgery without BTS [39]. However, its benefit in RMCO remains unclear. Rosander et al. also compared these two approaches and similarly found significant improvements in postoperative mortality and OS for left-sided disease, consistent with Veld et al., whereas no significant associations were observed in right-sided obstruction [27]. In our meta-analysis, decompression stoma likewise did not show any clear advantage over other initial management strategies and, conversely, ranked worst regarding the rate of stoma formation after resection. Taken together, the existing evidence supports decompression stoma as a BTS option only in left-sided obstruction, while current data do not support its use in RMCO.

A third BTS modality is decompression tube placement, although its evidence base remains limited as most available studies have relatively small sample sizes and originate mainly from East Asian countries. Nasal decompression tubes can reach the small bowel. However, they often do not work efficiently in severe obstruction due to an insufficient length, particularly for decompression of the cecum. In addition, transanal decompression tubes require more complicated colonoscopic skills as compared to left-sided cases, which limits their applicability. Even if decompression tube placement alone may be insufficient, combining it with other management strategies might provide additional benefits. Boeding et al. also reported that the advantage of optimization in RMCO, which is defined as postponing surgery to allow time for preoperative assessment of the patient’s health condition, identification of comorbidities, and optimization of medical status, may contribute to better outcomes regardless of the specific type of BTS approach used [40].

This study has several limitations. First, all included studies were non-randomized and retrospective in design, which increases the risk of confounding and selection bias. Although PSM data were used when available, residual confounding cannot be excluded. In addition, variations in patient characteristics and treatment selection across studies may have affected the robustness and comparability of the treatment nodes within the network. Second, definitions and classifications varied across studies. Some studies did not specify the exact type of BTS, resulting in the presence of an “any-BTS” node in addition to specific BTS nodes, and the decompression tube node included both nasal and transanal tubes. In addition, although we restricted inclusion to studies enrolling patients with potentially curable RMCO, eligibility criteria such as tumor stage, tumor location, and the definition of “potentially curable” varied across studies. Outcome definitions, including postoperative mortality, also varied across studies. These variations may have introduced clinical heterogeneity and could not be fully eliminated. Third, survival outcomes were synthesized using OS rates at the longest reported follow-up rather than time-to-event measures such as hazard ratios, as these were not consistently reported across studies. Because follow-up durations varied among studies, differences in survival rates may reflect variations in follow-up length rather than true differences in treatment effect. Consequently, survival findings should be interpreted with caution. Finally, because only a limited number of studies reported postoperative recurrence, the available data were insufficient to perform a meaningful analysis. As a result, the impact of different treatment strategies on recurrence could not be evaluated and should be investigated in future studies.

Despite such limitations, this systematic review and network meta-analysis clearly reaffirm that RMCO carries a postoperative mortality exceeding 5%, with nearly all deaths occurring after immediate surgery. This finding is clinically relevant. Stenting as a bridge-to-surgery (BTS) approach was associated with a lower postoperative mortality as compared to other treatment strategies included in the analysis. However, the feasibility of endoscopic stenting may be limited in some emergency settings due to the availability of experienced endoscopists and institutional resources. As a result, while stenting may offer major clinical benefits, its implementation as a first-line strategy should be considered in the context of local expertise, resource availability, and patient-specific factors. Additionally, particular attention should be paid to the risk of stent-related complications. These findings underscore the importance of selecting the initial management strategy based on each patient’s condition and disease presentation in RMCO.

## Conclusion

In this systematic review and Bayesian network meta-analysis comparing multiple initial management options for potentially curable RMCO, including immediate surgery, stent, decompression stoma, and decompression tube as a BTS approach, no significant differences were observed among treatment groups in terms of overall postoperative morbidity or OS. However, stenting was associated with a significantly lower postoperative mortality and a lower rate of stoma formation after resection as compared to immediate surgery without BTS. These findings may help guide individualized treatment selection and support shared decision-making in the management of RMCO. Most importantly, when the risk of postoperative mortality is high, stenting may represent a reasonable alternative to immediate surgery, particularly when appropriate expertise and resources are available.

## Supplementary Information

Supplementary Material 1. Supplementary Figure 1: Results of inconsistency testing using the node-splitting model. (A) Overall postoperative morbidity. (B) Postoperative mortality. (C1) Stoma formation after resection in the primary analysis, in which significant inconsistency was observed. (C2) Stoma formation after resection in the sensitivity analysis excluding the study containing the decompression tube group, in which the inconsistency disappeared. (D) Overall survival rate (BTS, bridge to surgery; IS, immediate surgery without BTS; Stent, stent as BTS; Stoma, decompression stoma as BTS; Tube, nasal/transanal decompression tube as BTS; CrI, credible interval).

Supplementary Material 2. Supplementary Figure 2: Forest plots, pairwise comparisons, and the SUCRA-based treatment hierarchy derived from the Bayesian network meta-analysis of initial management strategies for potentially curable right-sided malignant colonic obstruction for severe postoperative morbidity. (BTS, bridge to surgery; IS, immediate surgery without BTS; Stent, stent as BTS; Stoma, decompression stoma as BTS; Tube, nasal or transanal decompression tube as BTS; CrI, credible interval; SUCRA, surface under the cumulative ranking curve, where higher SUCRA values indicate a better treatment ranking.)

Supplementary Material 3.

## Data Availability

The data can be obtained from the corresponding author upon reasonable request.
