# Supplementary material for: Initial management strategies for right-sided malignant colonic obstruction: a systematic review and bayesian network meta-analysis
Source: World J Surg Oncol. 2026 Apr 2;24:202. doi: 10.1186/s12957-026-04303-9 (PMC13169969; doi:10.1186/s12957-026-04303-9)
Supplement: Supplementary file 3 — Supplementary Material 3. [file 12957_2026_4303_MOESM3_ESM.docx]

**Supplementary Table 1:** Search strategies for each database

| Database | Date of last search | Search strategy |
| --- | --- | --- |
| PubMed | 2025-10-31 | (("Colorectal Neoplasms"[Mesh] OR "colon cancer"[tiab] OR "colorectal cancer"[tiab]) AND (obstruction*[tiab] OR obstructive[tiab] OR ileus[tiab]) AND ("right-sided"[tiab] OR "right sided"[tiab] OR "right and"[tiab] OR proximal[tiab] OR cecum[tiab] OR "ascending colon"[tiab]) AND ((stent*[tiab] OR "self-expandable metallic stent"[tiab] OR SEMS[tiab] OR "bridge to surgery"[tiab]) OR (stoma[tiab] OR ileostom*[tiab] OR colostom*[tiab] OR diversion[tiab]) OR decompression[tiab] OR (surgery[tiab] OR colectomy[tiab] OR resection[tiab] OR "right hemicolectomy"[tiab])) AND (randomized controlled trial[pt] OR random*[tiab] OR trial[ti] OR comparative[tiab] OR compare*[tiab] OR "comparative study"[pt] OR cohort[tiab] OR retrospective[tiab] OR groups[tiab] OR propensity[tiab] OR "case-control"[tiab] OR observational[tiab])) |
| Embase | 2025-10-31 | ('colorectal tumor'/exp OR 'colon cancer'/exp OR 'colorectal cancer'/exp OR "colon cancer":ti,ab OR "colorectal cancer":ti,ab)  AND (obstruction*:ti,ab OR obstructive:ti,ab OR ileus:ti,ab) AND ("right-sided":ti,ab OR "right sided":ti,ab OR "right and":ti,ab OR proximal:ti,ab OR cecum:ti,ab OR "ascending colon":ti,ab) AND ((stent*:ti,ab OR "self-expandable metallic stent":ti,ab OR SEMS:ti,ab OR "bridge to surgery":ti,ab) OR (stoma:ti,ab OR ileostom*:ti,ab OR colostom*:ti,ab OR diversion:ti,ab) OR decompression:ti,ab OR (surgery:ti,ab OR colectomy:ti,ab OR resection:ti,ab OR "right hemicolectomy":ti,ab)) AND (random*:ti,ab OR trial:ti OR comparative:ti,ab OR compare*:ti,ab OR "comparative study":ti,ab OR cohort:ti,ab OR retrospective:ti,ab OR groups:ti,ab OR propensity:ti,ab OR "case-control":ti,ab OR observational:ti,ab) |
| Cochrane Library | 2025-10-31 | (("Colorectal Neoplasms" OR "colon cancer" OR "colorectal cancer") AND (obstruction* OR obstructive OR ileus) AND ("right-sided" OR "right sided" OR "right and" OR proximal OR cecum OR "ascending colon") AND ((stent* OR "self-expandable metallic stent" OR SEMS OR "bridge to surgery") OR (stoma OR ileostom* OR colostom* OR diversion) OR decompression OR (surgery OR colectomy OR resection OR "right hemicolectomy")) AND (randomized OR random* OR trial OR comparative OR compare* OR cohort OR retrospective OR groups OR propensity OR "case-control" OR observational)) |

**Supplementary Table 2:** Bayesian Model Specifications and Diagnostics

| Component | Specification |
| --- | --- |
| Analysis framework | Bayesian network meta-analysis (consistency model) |
| Effect model | Random-effects |
| Software | MetaInsight v6.4.0 (CRSU; NIHR project code: NIHR153934) |
| Computational backend | R packages: **gemtc** (v0.8-2), **BUGSnet** (v1.0.3), **bnma** (v1.6.0) |
| Likelihood | Binomial (binary outcomes); Gaussian (continuous outcomes) |
| Effect measures | RR for binary outcomes; MD for continuous outcomes |
| Number of chains | 4 |
| Burn-in iterations | 5,000 |
| Sampling iterations | 20,000 per chain |
| Thinning factor | 1 |
| Prior for relative treatment effects | Normal (0, 2940.5) |
| Prior for intercepts | Normal (0, 2940.5) |
| Prior for heterogeneity SD (τ) | Uniform (0, 3.6) |
| Convergence diagnostics | Gelman convergence assessment plots, trace plots, posterior density plots |
| Model fit diagnostics | Residual deviance from NMA model; residual deviance from UME inconsistency model |
| Influence diagnostics | Per-arm residual deviance plots; leverage plots |
| Inconsistency assessment | Node-splitting approach |
| DIC-based model comparison | Not performed |

CRSU, Complex Reviews Support Unit; NIHR, National Institute for Health and Care Research; RR, risk ratio; MD, mean difference; SD, standard deviation; τ, between-study heterogeneity standard deviation; NMA, network meta-analysis; UME, unrelated mean effects; DIC, Deviance Information Criterion.

**Supplementary Table 3:** Baseline characteristics of patients in the included studies

| Author/Year | Age (years) | Sex (male) | ASA-PS ≧ III | Stage IV |
| --- | --- | --- | --- | --- |
| Amelung 2016 | 71.2 | 902/1,860 (48.5%) | 633/1,830 (34.6%) | 506/1,769 (28.6%) |
| Amelung 2017 | 70.3 | 49/110 (44.5%) | 15/110 (13.6%) | 34/110 (30.9%) |
| Boeding 2023 | 73.0 | 262/525 (49.9%) | 146/417 (35.0%) | 150/524 (28.6%) |
| Hotta 2012 | NR | 74/138 (53.6%) | NR | 0 |
| Huang 2024 | 59.2 | 48/95 (50.5%) | NR | 11/95 (11.6%) |
| Ji 2017 | 65.0 | 15/39 (38.5%) | 5/39 (12.8%) | 15/39 (38.5%) |
| Kim 2023 | 66.4 | 64/167 (38.3%) | 56/167 (33.5%) | 9/167 (5.4%) |
| Kye 2016 | 70.3 | 35/74 (47.3%) | 11/74 (14.9%) | 0 |
| Li 2020 | 66.7 | 53/107 (49.5%) | NR | 14/107 (13.1%) |
| Lockhorst 2025 | 72.0 | 34/62 (54.8%) | 26/53 (49.1%) | 14/60 (23.3%) |
| Morita 2019 | 71.6 | 25/68 (36.8%) | 6/68 (8.8%) | 22/68 (32.4%) |
| Park 2023 | 72.3 | 40/72 (55.6%) | 21/72 (29.2%) | 0 |
| Rosander 2021 | 75.4 | 326/751 (43.4%) | 349/735 (47.5%) | 0 |
| Sakamoto 2020 | NR | 1,500/3,000 (50%) | NR | 688/3,000 (22.9%) |
| Suzuki 2019 | 67.5 | 19/40 (47.5%) | NR | 6/40 (15%) |
| Takahashi 2024 | 72.3 | 17/28 (60.7%) | NR | 0 |
| van den Berg 2014 | 71.0 | 18/34 (52.9%) | NR | 11/34 (32.4%) |
| Zeng 2021 | 65.8 | 166/294 (56.5%) | 161/294 (54.8%) | 0 |

ASA-PS, American Society of Anesthesiologists physical status; NR, not reported.

**Supplementary Table 4:** Potential effect modifiers across treatment nodes

|  | Stent | Stoma | Tube | IS | Any BTS |
| --- | --- | --- | --- | --- | --- |
| Number of studies | 14 | 4 | 3 | 15 | 2 |
| Sample size | 1.903 | 144 | 49 | 5.053 | 56 |
| Sex (male) | 945/1.886 (50.1%) | 76/142 (53.2%) | 24/41 (58.5%) | 2.209/4.586 (48.2%) | 11/30 (36.7%) |
| Age (years) | 66.9 | 68.8 | 70.0 | 71.6 | 72.0 |
| ASA-PS ≥ III | 92/273 (33.7%) | 41/142 (28.9%) | NR | 1.098/2912 (37.7%) | 17/30 (56.7%) |
| Stage IV | 386/1.885 (20.5%) | 15/143 (10.5%) | 1/49 (2.0%) | 980/5.013 (19.5%) | 14/55 (25.5%) |

ASA-PS, American Society of Anesthesiologists physical status; NR, not reported; BTS, bridge to surgery; IS, immediate surgery without BTS.

**Supplementary Table 5:** Surface Under the Cumulative Ranking Curve (SUCRA) values for each treatment (higher SUCRA scores indicate a better ranking).

|  | Overall postoperative morbidity | Postoperative mortality | Stoma formation after resection | Overall survival rate |
| --- | --- | --- | --- | --- |
| Any BTS | 76.09 | 81.50 | 44.11 | 45.38 |
| IS | 17.86 | 8.05 | 48.80 | 22.87 |
| Stent | 58.56 | 73.43 | 92.74 | 47.03 |
| Stoma | 64.47 | 37.02 | 28.65 | 45.46 |
| Tube | 33.03 | NA | 35.70 | 89.26 |

BTS, bridge to surgery; IS, immediate surgery without BTS; Stent, stent as BTS; Stoma, decompression stoma as BTS; Tube, nasal/transanal decompression tube as BTS; NA, not applicable.
